# Chronic Urticaria-Associated Syndromes: Is Andersen–Tawil Syndrome One of Them?

**DOI:** 10.3390/genes17080976

**Published:** 2026-08-19

**Authors:** Vedrana Bulat, Lucija Zanze, Mirta Peček, Dajana Smoljan-Filipović, Katarina Dragun, Liborija Lugović-Mihić

**Affiliations:** 1 Department of Dermatovenereology, Sestre Milosrdnice University Hospital Center, Vinogradska cesta 29, 10000 Zagreb, Croatia; veckybulat@gmail.com (V.B.); dajanas2111@gmail.com (D.S.-F.); dragunkatarina1@gmail.com (K.D.); 2 Family Physician Office, 10000 Zagreb, Croatia; lucijazanze15@gmail.com; 3 Lipik Special Hospital for Medical Rehabilitation, 34551 Lipik, Croatia; mirta.pec@gmail.com; 4 Chair of Dermatovenerology, University of Zagreb School of Dental Medicine, 10000 Zagreb, Croatia

**Keywords:** chronic urticaria, syndrome, autoinflammatory syndrome, Andersen–Tawil syndrome, cryopyrin-associated periodic syndrome, periodic fever syndrome, Schnitzler syndrome, hypereosinophilic syndrome, Wells syndrome, Sjögren syndrome

## Abstract

The spectrum of syndromes associated with chronic urticaria (CU) is broad, ranging from monogenic autoinflammatory diseases (a single gene defect drives disease through dysregulated innate immunity) to multifactorial and acquired conditions (urticaria arises as part of a broader, polygenic or immune-mediated systemic process). Monogenic autoinflammatory conditions presenting with urticaria include cryopyrin-associated periodic syndromes (CAPS)—encompassing familial cold autoinflammatory syndrome (FCAS), Muckle–Wells syndrome (MWS), and neonatal-onset multisystem inflammatory disease (NOMID/CINCA) and the broader group of familial cold urticarias. Monogenic conditions with well-characterized gain-of-function NLR family pyrin domain containing 3 (*NLRP3*) variants present with recurrent hives, a cardinal feature associated with recurrent fevers. Multifactorial and acquired autoinflammatory or immune-mediated conditions include Schnitzler syndrome, adult-onset Still’s disease, hypereosinophilic syndrome, Gleich syndrome, Wells syndrome, and Sjögren syndrome. Multifactorial conditions, including Schnitzler syndrome and Still’s disease, manifest similarly, with CU as an initial sign and a shared pathogenic mechanism of innate immune dysregulation, with interleukin-1β playing a central, pro-inflammatory role as a major pyrogen. Here, we also present a female patient with clinically established Andersen–Tawil syndrome (ATS) who developed recurrent hives on her extremities within minutes after vigorous exercise. To our knowledge, this is the first report of a co-occurrence of CU and ATS. The hives were successfully attenuated by oral intake of effervescent potassium chloride during physical activity. This observation raises the possibility that chronic inducible urticaria may represent a previously unrecognized cutaneous ATS manifestation and suggests a potential pathophysiological link between potassium-channel dysfunction and mast cell activation. Distinguishing whether hives are an isolated symptom or part of a broader syndrome is critically important, as it might be crucial for the patient outcome.

## 1. Introduction

**Figure 1:**
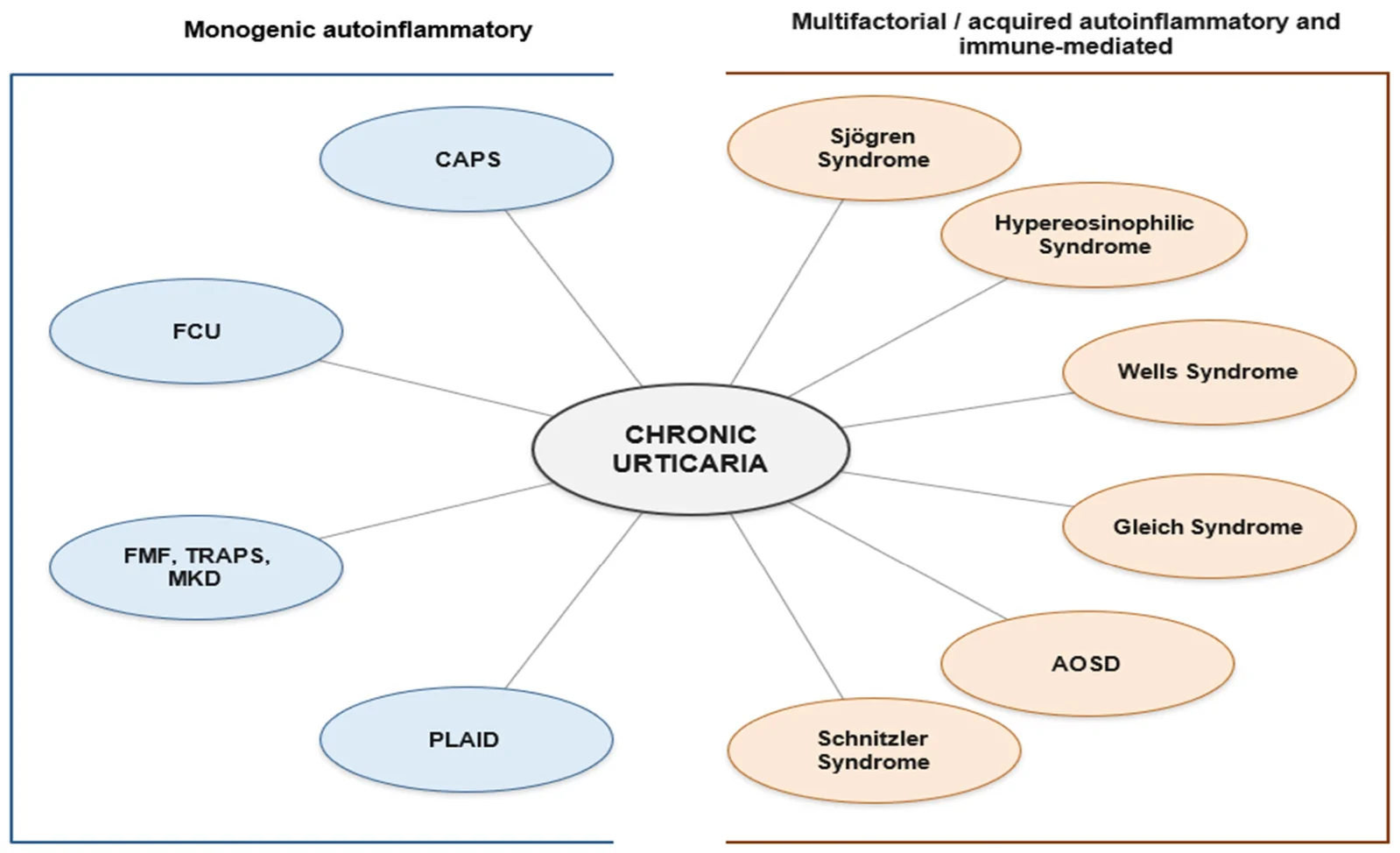
Schematic overview of monogenic autoinflammatory (blue) and multifactorial/acquired autoinflammatory and immune-mediated (orange) syndromes associated with chronic urticaria. CAPS—cryopyrin-associated periodic syndromes; FCU—familial cold urticaria; FMF—familial Mediterranean fever; TRAPS—TNF receptor-associated periodic syndrome; MKD—mevalonate kinase deficiency; PLAID—*PLCG2*-associated antibody deficiency and immune dysregulation; AOSD—adult-onset Still’s disease.

Chronic urticaria (CU) is classically understood as a mast cell-driven, histaminergic disorder; however, emerging evidence has established that there is a clinically significant subset of CU cases where an underlying systemic autoinflammatory process is at work rather than a primary allergic or idiopathic one [1]. The association between CU and genetic factors, including both monogenic and multifactorial autoinflammatory conditions, has been confirmed in the medical literature, repositioning CU as a potential early or dominant cutaneous manifestation of systemic autoinflammatory disease (Figure 1) [2].

The spectrum of autoinflammatory syndromes associated with CU is broad. Monogenic conditions with well-characterized gain-of-function or loss-of-function mutations—most notably cryopyrin-associated periodic syndromes (CAPS), caused by mutations in the gene *NLR family pyrin domain containing 3* (*NLRP3)*—present with recurrent hives as a cardinal feature. Multifactorial conditions, including Schnitzler syndrome and Still’s disease, manifest similarly, with CU as an initial sign. Across this spectrum, the shared pathogenic mechanism is innate immune dysregulation (abnormalities of the inflammasome). The inflammasome is a macromolecular complex that senses exogenous danger signals (cold and PAMPs, or pathogen-associated molecular patterns) and endogenous danger signals (DAMPs—damage-associated molecular patterns) and activates caspase-1 and, ultimately, interleukin-1β (IL-1β) and its central pro-inflammatory role [2,3].

A unifying histopathological finding in these autoinflammatory urticarias is neutrophilic urticarial dermatosis—a pattern in which neutrophils accumulate around small dermal vessels and between collagen bundles, with fragmentation of neutrophil nuclei, but without the vessel-wall damage that defines true vasculitis or the tissue swelling typical of classic mast cell-mediated urticaria. This distinct pattern carries direct diagnostic significance. Depending on the underlying syndrome, associated laboratory findings can include neutrophilic leukocytosis and elevated acute-phase reactants such as erythrocyte sedimentation rate (ESR) and CRP [2,4].

Recognizing CU as a potential manifestation of an underlying autoinflammatory syndrome has critical clinical implications: CU refractory to standard antihistamine therapy, particularly when accompanied by systemic features such as fever, joint or muscle pain, organ involvement (serosal, hepatosplenic, ocular, or neurological), and elevated inflammatory markers, warrants a structured diagnostic workup—including histopathological examination, serological assessment, and targeted genetic testing—to enable proper therapeutic intervention [2].

This article is a narrative review with an illustrative case included, thus in total involving a review with a diagnostic overview, accompanied by a detailed single-patient case report. When selecting articles/publications for this review, we analyzed all references published in English to date, up to June 2026, in the PubMed, Scopus, and Web of Science databases using the medical terms/keywords “chronic urticaria”; “syndrome”; “autoinflammatory syndrome”; “Andersen-Tawil syndrome”; “cryopyrin-associated periodic syndrome”; “periodic fever syndrome”; “Schnitzler syndrome”; “hypereosinophilic syndrome”; “Wells syndrome”; and “Sjögren syndrome”. We limited our search to articles specifically related to the spectrum of syndromes associated with CU.

## 2. Features of Common Syndromes Associated with Chronic Urticaria

Syndromes associated with CU span a spectrum ranging from monogenic autoinflammatory diseases, in which a single gene defect drives disease through dysregulated innate immunity, to multifactorial and acquired conditions in which urticaria arises as part of a broader, polygenic or immune-mediated systemic process [2].

Monogenic autoinflammatory conditions presenting with urticaria include cryopyrin-associated periodic syndromes (CAPS or cryopyrinopathies)—encompassing familial cold autoinflammatory syndrome (FCAS), Muckle–Wells syndrome (MWS), and neonatal-onset multisystem inflammatory disease (NOMID/CINCA)—and the broader group of familial cold urticarias (FCUs), which includes familial atypical cold urticaria (FACU), *NLRP3*-, *NLRP12*-, *NLRC4*-, and *PLCG2*-related FCAS subtypes, FXII-associated cold autoinflammatory syndrome (FACAS), and familial predisposed acquired cold urticaria (FP-ACU) [5,6]. Additional monogenic periodic fever syndromes relevant as differential diagnoses include familial Mediterranean fever (FMF), TNF receptor-associated periodic syndrome (TRAPS), mevalonate kinase deficiency (MKD) and *PLCG2*-associated antibody deficiency and immune dysregulation (PLAID) [7,8,9]. Multifactorial and acquired autoinflammatory or immune-mediated conditions include Schnitzler syndrome, adult-onset Still’s disease, hypereosinophilic syndrome, Gleich syndrome (episodic angioedema with eosinophilia), Wells syndrome (eosinophilic cellulitis), and Sjögren syndrome [10,11,12,13,14].

Across this spectrum, the shared pathogenic thread is dysregulation of the innate immune system—“autoinflammation”—as distinct from the adaptive immune dysfunction that characterizes classic autoimmunity [5]. Excessive activation of the interleukin-1β (IL-1β) pathway, frequently via inflammasome complexes such as *NLRP3*, *NLRP12*, and *NLRC4*, is considered the primary underlying mechanism in most monogenic forms [3,5,7].

Clinically, these conditions typically present with recurrent inflammatory episodes featuring fever, which is very common, elevated acute-phase reactants, and skin lesions occurring without infectious or autoimmune cause. Cutaneous involvement varies in pattern: hives are a key manifestation, particularly in cryopyrinopathies, typically appearing early in life; periodic fever syndromes commonly present with erythematous macules and urticaria; FMF more often presents with erysipelas-like lower-limb lesions. Additional systemic features may include abdominal pain, myalgia, serositis, ocular involvement, amyloidosis, and neurological abnormalities [7,15].

Diagnosis relies primarily on clinical presentation, supported by genetic analysis, where a therapeutic response to IL-1 inhibition may also support a diagnosis. For skin biopsies, the characteristic histopathological finding is a perivascular/interstitial neutrophil-rich infiltrate consistent with neutrophilic urticarial dermatosis. Given this broad clinical spectrum, management of these conditions requires a multidisciplinary approach involving dermatology, rheumatology, pediatrics, internal medicine, and infectious disease specialties [4,7,15].

In clinical practice, it is sometimes difficult to recognize and differentiate the various conditions that involve urticaria/hives. Despite this, a few prominent types can be distinguished based on their specific features: (1.) true chronic spontaneous urticaria (CSU) and chronic inducible urticaria; (2.) neutrophilic urticarial dermatoses associated with autoinflammation; (3.) eosinophilic, vasculitic, and hematologic urticaria-like eruptions; (4.) autoimmune diseases that may coexist with urticaria without constituting a defined “urticaria syndrome” (Table 1). When differentiating these categories/groups, lesion duration, physical trigger testing, systemic “red flag” symptoms, and skin biopsy findings need to be considered. Thus, when evaluating a specific patient, the following information must be taken into account: the duration of individual skin lesions (under or over 24 h); whether any systemic symptoms (fever, joint pain) are present; results from any blood tests or skin biopsies [16,17].

## 3. Monogenic Autoinflammatory Conditions

This group involves conditions in which a single gene defect drives disease through dysregulated innate immunity.

### 3.1. Cryopyrin-Associated Periodic Syndromes (CAPS; FCAS, OMIM #120100; Muckle–Wells Syndrome, OMIM #191900; NOMID/CINCA, OMIM #607115)

CAPS is a rare autoinflammatory condition caused by gain-of-function mutations in the *NLRP3* gene, which encodes cryopyrin that forms a complex that activates caspase 1, manifesting with urticaria (hives), recurrent fever episodes, arthralgia, and systemic inflammation. CAPS is primarily associated with gain-of-function *NLRP3* variants. The syndrome comprises three clinically overlapping autosomal dominant syndromes: familial cold-induced autoinflammatory syndrome (FCAS), Muckle–Wells syndrome (MWS), and neonatal-onset multisystem inflammatory disease/chronic infantile neurologic cutaneous and articular syndrome (NOMID/CINCA). Episodes may be triggered by cold exposure, infection, fatigue, stress, trauma, or sleep deprivation [3,18]. Urticaria is the cardinal and earliest cutaneous feature across the entire CAPS spectrum, making it a key entry point for diagnostic suspicion in patients with cold-triggered or recurrent hives accompanied by systemic inflammatory signs.

### 3.2. Familial Cold Urticarias (FCUs; Multiple OMIM Phenotypes, Including FCAS1 OMIM #120100, FCAS2 OMIM #611762, FCAS4 OMIM #616115, PLAID OMIM #614878)

FCU represents a group of rare hereditary disorders triggered by cold exposure, with pathogenesis involving aberrant inflammasome activity and dysfunction of the kallikrein–kinin pathway. Recognized subtypes include familial atypical cold urticaria (FACU), familial cold autoinflammatory syndromes (FCAS, including *NLRP3*-related FCAS [OMIM #120100], *NLRP12*-related FCAS2 [OMIM #611762], *NLRC4*-related FCAS4 [OMIM #616115], and *PLCG2*-associated FCAS3/PLAID [OMIM #614468/#614878]), FXII-associated cold autoinflammatory syndrome (FACAS), and familial predisposed acquired cold urticaria (FP-ACU). Genetically, gain-of-function mutations in *NLRP3*, *NLRP12*, and *NLRC4* drive excessive inflammasome activation; in-frame deletions in *PLCG2* cause temperature-dependent dysregulation of immune signaling; and heterozygous *F12* variants link contact-system activation to inflammatory cascades. Integration of cold stimulation testing, inflammatory biomarker assessment, and targeted genetic sequencing enables molecular stratification of patients [6]. This group is directly relevant to CU because cold-induced urticaria is frequently the presenting, and most clinically prominent, feature, often preceding recognition of the underlying systemic autoinflammatory disorder.

### 3.3. Familial Mediterranean Fever (OMIM #249100), Tumor Necrosis Factor Receptor-Associated Periodic Syndrome (TRAPS; OMIM #142680), and Mevalonate Kinase Deficiency (MKD; OMIM #260920)

FMF, TRAPS, and MKD are classical hereditary periodic fever syndromes grouped within the autoinflammatory disease spectrum on the basis of shared IL-1β-driven inflammatory mechanisms, even though their primary cutaneous manifestations—erysipelas-like lesions in FMF, migratory erythematous skin plaques in TRAPS, and polymorphic rash (urticarial or maculopapular) in MKD—differ from classic urticaria. Their inclusion in this group is principally relevant in the differential diagnostic workup of patients presenting with recurrent fever and urticarial-type skin lesions in whom standard antihistamine therapy has failed, as these syndromes may clinically overlap with, or be mistaken for, autoinflammatory urticaria before a definitive genetic diagnosis is established [7,19].

### 3.4. PLCG2-Associated Antibody Deficiency and Immune Dysregulation (PLAID, OMIM #614878)

*PLCG2*-associated antibody deficiency and immune dysregulation (PLAID) is a rare, autosomal, dominantly inherited disease caused by heterozygous genomic deletions in *PLCG2*, which encodes phospholipase Cγ2, a key signaling molecule in immune cells. It is characterized almost universally by cold-induced urticaria present from infancy, typically triggered by evaporative cooling rather than direct contact with cold objects. The mutant *PLCG2* enzyme renders mast cells spontaneously active at lower skin temperatures even without antigen exposure. *PLCG2* mutations give rise to a broad immunological spectrum, ranging from phenotypes dominated by immunodeficiency to those in which autoinflammatory features predominate. Beyond urticaria, patients may develop additional variable features including granulomatous skin lesions, recurrent sinopulmonary infections, low serum immunoglobulins (IgM, IgA), and autoimmune phenomena such as thyroiditis or vitiligo, with up to two-thirds of patients showing positive antinuclear antibodies [8,9].

The conditions above, characterized by various genetic and clinical features, are outlined in Table 2 [2,8,20,21,22,23,24,25,26,27,28,29,30,31,32,33,34].

## 4. Multifactorial/Acquired Autoinflammatory and Immune-Mediated Conditions

This group involves a handful of conditions, in which urticaria arises as part of a broader, polygenic or immune-mediated systemic process.

### 4.1. Schnitzler Syndrome

Schnitzler syndrome is an autoinflammatory disease characterized by a triad of recurrent urticaria, monoclonal IgM gammopathy, and systemic inflammation associated with arthralgia and lymphadenopathy, accompanied by recurrent fever, joint and bone pain, and atypical bone remodeling (osteosclerosis) with leukocytosis and elevated C-reactive protein (CRP). Pathogenetically, aberrant activation of the NLRP3 inflammasome drives IL-1 production, with additional involvement of IL-6 and IL-18. Mosaicism in myeloid cells for somatic mutations in the *NLRP3* gene has been reported. Treatment relies on IL-1 inhibitors (anakinra, rilonacept, canakinumab), with tocilizumab (IL-6 receptor inhibitor) and ibrutinib (Bruton’s tyrosine kinase inhibitor) emerging as promising alternatives. Owing to its rarity, diagnosis remains challenging in clinical practice; notably, incomplete “Schnitzler-like” variants lacking monoclonal gammopathy may still respond to IL-1 blockade and warrant diagnostic consideration [10,35]. Recurrent urticaria is the defining feature, making Schnitzler syndrome a critical part of any CU workup for systemic inflammation and monoclonal gammopathy.

### 4.2. Adult-Onset Still’s Disease (AOSD)

Adult-onset Still’s disease (AOSD) is a non-hereditary, polygenic systemic autoinflammatory condition characterized by four major features: a spiking fever, an evanescent salmon-pink maculopapular rash, joint involvement (arthralgia or arthritis), and leukocytosis, predominantly neutrophilic. Pathogenesis centers on strong innate immune activation mediated by IL-1β, leading to overproduction of cytokines IL-6, IL-8, IL-17, IL-18 and tumor necrosis factor (TNF) [36]. Treatment predominantly includes systemic corticosteroids, antirheumatic drugs, and targeted biologic therapy—IL-1 inhibitors (anakinra, canakinumab) and the anti-IL-6 agent tocilizumab [13]. Hives typically coincide with fever spikes and may precede recognition of the full systemic syndrome. They appear mainly on the proximal limbs or trunk, a cutaneous hallmark linking this condition directly to the urticaria-associated autoinflammatory spectrum [36].

### 4.3. Hypereosinophilic Syndrome

Hypereosinophilic syndrome is a heterogeneous group of disorders characterized by chronic eosinophil overproduction leading to tissue infiltration and damage. Cutaneous manifestations may include recurrent urticaria, although angioedema and erythroderma are more typical. Diagnosis is based on sustained, unexplained peripheral eosinophilia ≥ 1500/µL, evidence of eosinophil-driven organ dysfunction, and exclusion of secondary causes including parasitic infection and allergy. Its relevance to CU lies in the overlap of recurrent urticarial lesions with eosinophil-driven mechanisms distinct from classic mast-cell or IL-1-driven pathways, broadening the differential diagnosis of antihistamine-refractory urticaria [11,14,37].

### 4.4. Gleich Syndrome (Episodic Angioedema with Eosinophilia)

Gleich syndrome is characterized by recurrent episodic angioedema, urticaria, fever and weight gain occurring with marked eosinophilia. Diagnostic uncertainty is frequently seen in clinical practice—the median time from first flare to diagnosis has been reported at five years. Pathogenetically, mediators and cytokines released from circulating eosinophils driven by T lymphocytes are thought to directly trigger cutaneous mast-cell degranulation, releasing histamine and other mediators that produce hives and erythema [14,38]. When the cyclical urticaria–angioedema–eosinophilia pattern is observed in recurrent CU with associated eosinophilia, Gleich syndrome should be considered, particularly when angioedema and weight gain accompany flares.

### 4.5. Wells Syndrome (Eosinophilic Cellulitis)

Wells syndrome is a rare dermatosis characterized by acute, recurrent, pruritic, erythematous, and edematous urticaria-like lesions that can evolve into erythematous pruritic plaques with a granuloma annulare-like appearance. Some lesions can evolve into nodules and bullae mimicking vasculitis. It is thought to be a type IV hypersensitivity reaction caused by triggers such as insect bites, infections, medications, malignancies, and eosinophilic systemic diseases. Diagnosis relies on the clinical picture together with histopathological features—typically dermal edema, dermal eosinophilic infiltration, and free eosinophilic granules coating collagen fibers—that can vary over the course of the disease. Peripheral eosinophilia may additionally be present during acute episodes [11,39]. The initial urticarial presentation of Wells syndrome is a key reason it enters the differential diagnosis of CU, particularly when lesions evolve atypically or fail to respond to antihistamines.

### 4.6. Sjögren Syndrome

Sjögren syndrome is a chronic autoimmune condition primarily characterized by fatigue, joint and muscle pain, and severe exocrine gland dysfunction causing dryness, with diverse localized and systemic manifestations. It is considered a multifactorial disease in which an underlying genetic predisposition, epigenetic mechanisms, and environmental factors contribute to disease development. The strongest genetic association lies within the HLA gene region, while the non-HLA genes *IRF5* and *STAT4* show consistent, smaller-effect associations across multiple ethnicities [12]. Patients with Sjögren syndrome more frequently experience allergic disorders and recurrent or chronic hives. In some cases, CU or recurrent severe allergic reactions may represent an atypical first presentation, with intractable urticaria and joint pain occasionally serving as early indicators of an underlying autoimmune condition [40].

## 5. Urticaria Potentially Related to Vasculitic and Hematologic Disorders

In addition to primary dermatological disorders, numerous systemic conditions may present with hives (urticaria). Distinguishing these systemic urticarial syndromes from conventional urticaria requires attention to several clinical features: lesions may include not only hives but also papules, vesicles, hemorrhagic lesions, necrosis, and crusts; lesions are frequently accompanied by systemic manifestations such as fever, fatigue, and arthralgia; and individual lesions typically persist for longer than 24–36 h, exhibit a bilateral and symmetrical distribution, and often resolve with residual hyperpigmentation and bruising, in contrast to the transient, non-scarring hives of ordinary urticaria, which characteristically resolve within 24 h and leave no residual marks [41].

Urticarial vasculitis (UV) is a rare small-vessel leukocytoclastic vasculitis defined by recurrent urticaria persisting longer than 24 h, with histopathological features of leukocytoclastic vasculitis, and represents an important differential diagnosis in the workup of systemic urticarial syndromes. Lesions are often painful or burning rather than pruritic, and heal with residual post-inflammatory hyperpigmentation, in contrast to the transient, non-scarring hives of ordinary urticaria [41,42].

The characteristic histopathological finding is leukocytoclastic vasculitis of the small dermal vessels, with a neutrophil-rich perivascular infiltrate, neutrophil fragmentation (“leukocytoclasia”), nuclear dust, erythrocyte extravasation, and fibrin deposition within and around vessel walls [42].

UV is an immune complex-mediated type III hypersensitivity that is classified into two subtypes based on complement status: normocomplementemic UV (NUV), which typically lacks significant systemic involvement and carries a more favorable prognosis, and hypocomplementemic UV (HUV), which shows a tendency toward more severe multisystem involvement—frequently affecting the joints, kidneys, lungs, and gastrointestinal or neurologic systems. NUV is most often idiopathic, while HUV may be associated with systemic diseases (SLE, primary Sjögren’s syndrome, monoclonal gammopathy), medications and hematologic disorders. The most frequent laboratory abnormalities in idiopathic UV are an elevated ESR and decreased serum complement levels. The majority of HUV patients are antinuclear antibody (ANA)-positive [42].

A recent expert-consensus decision-making algorithm on UV treatment proposes a stepwise approach based on disease severity, assessed via the Urticarial Vasculitis Activity Score (UVAS7): patients with mild, skin-limited disease (UVAS7 ≤ 7) may be managed in a way similar to chronic urticaria treatment, with second-generation H1-antihistamines, omalizumab, or cyclosporine A, while patients with more severe disease (UVAS7 > 7)—particularly those with hypocomplementemic UV or an underlying systemic disease such as SLE, malignancy, or infection—typically require a multidisciplinary approach. Immunomodulatory options in more severe cases include systemic corticosteroids, dapsone, hydroxychloroquine, anti-IL-1 agents, and other steroid-sparing immunosuppressants, selected according to clinical presentation and safety profile [43].

Beyond urticarial vasculitis, hives may also occur as a cutaneous manifestation of several other primary systemic vasculitides—most notably eosinophilic granulomatosis with polyangiitis (EGPA, formerly Churg–Strauss syndrome), polyarteritis nodosa (PAN), and granulomatosis with polyangiitis (GPA, formerly Wegener granulomatosis). EGPA is a rare ANCA-associated vasculitis with eosinophilic and granulomatous features in which urticarial lesions may occur alongside more characteristic red flags including asthma, nasal polyposis, marked peripheral eosinophilia, polyneuropathy, and palpable purpura with eosinophilic infiltrates on a biopsy [41,44]. PAN is a rare necrotizing vasculitis of medium-sized vessels whose cutaneous involvement typically presents as palpable purpura, livedo reticularis, or subcutaneous nodules, with urticarial lesions representing a less common finding, reported in approximately 6% of patients [41,45]. GPA is an ANCA-associated granulomatous vasculitis classically affecting the upper respiratory tract, lungs, kidneys and skin. Urticarial eruptions are very rare and occur alongside palpable purpura and other vasculitic lesions such as papulonecrotic lesions, livedo, ulcers and digital infarcts [41,44].

## 6. Andersen–Tawil Syndrome Associated with Chronic Urticaria—A Hypothesis-Generating Observation? (OMIM #170390)

Andersen–Tawil syndrome (ATS) is an exceptionally rare autosomal dominant multisystem disease caused by dysfunction of potassium ion channels, with an estimated prevalence of less than 1 per 1,000,000 individuals [46]. First described by Ellen Andersen in 1971, and subsequently further delineated by Rabi Tawil, ATS is classically defined by a clinical triad of periodic paralysis, ventricular arrhythmias and characteristic dysmorphic features including craniofacial anomalies and digital abnormalities such as clinodactyly [47]. The clinical expression of ATS is highly variable and incomplete penetrance is common, making early detection challenging [48]. Despite advances in elucidating its cardiac and neuromuscular mechanisms, the broader systemic phenotype of ATS remains incompletely characterized [49]. Mutations in *KCNJ2* (potassium inwardly rectifying channel subfamily J member 2) and *KCNJ5* are the best-known genetic causes of ATS. *KCNJ2* (17q24.3) encodes the Kir2.1, an inwardly rectifying lipid-gated potassium channel, crucial for maintaining resting membrane potential in multiple tissues, including skeletal and cardiac muscle, and the brain [50]. Because Kir2.1 channels are expressed in multiple tissues, ATS may have broader systemic manifestations that remain incompletely characterized [49,50,51,52,53]. *KCNJ5* (11q24.3) encodes the Kir3.4 G-protein-activated potassium channel, which modulates inward potassium currents [54]. Approximately 60% of cases (ATS type 1, ATS1) are attributable to mutations in *KCNJ2*, which encodes the inward rectifier potassium channel subunit Kir2.1. More than 90 pathogenic variants have been identified, with most exerting dominant-negative loss-of-function effects that markedly reduce the inward rectifier potassium current [55]. A considerable proportion of mutations arise de novo, underscoring the genetic complexity of the disorder. An impaired potassium channel destabilizes the resting membrane potential in skeletal and cardiac muscle, predisposing affected individuals to episodic membrane inexcitability and electrical instability. The remaining cases, sometimes categorized as ATS type 2 (ATS2), are associated with other, or as yet unidentified, genetic substrates, suggesting that additional genes may contribute to the disorder.

**Figure 2:**
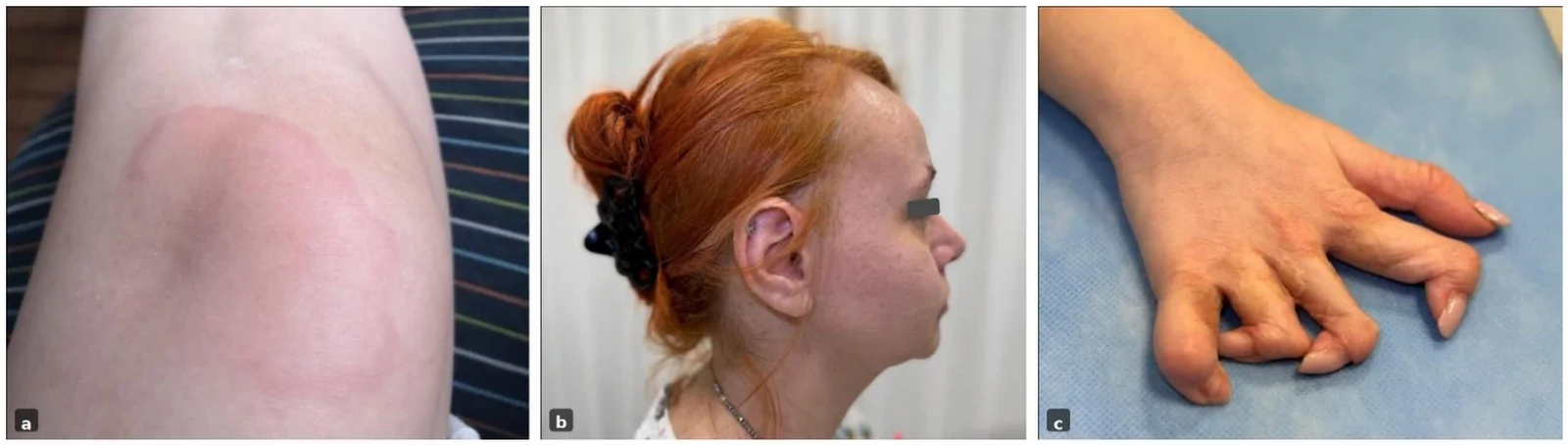
(**a**) Appearance of urticaria on her knee a few minutes after vigorous exercise.; (**b**) patient with small lower jaw (micrognathia) and a prominent forehead.; (**c**) clinodactyly of the fifth finger and camptodactyly after surgical separation of congenital syndactyly between the 2nd and 3rd fingers and the 3rd and 4th fingers.

Here, we report a patient with a clinical diagnosis of ATS, without molecular confirmation, who also experienced recurrent hives without angioedema (Table 3; Figure 2). This co-occurrence is presented as a hypothesis-generating observation and does not establish urticaria as a manifestation of ATS. The 28-year-old woman presented to our department in October 2020 with recurrent episodes of hives without angioedema. She reported the appearance of up to ten pruritic hives measuring less than 5 cm in diameter and lacking a surrounding pale halo, symmetrically distributed over the upper and lower extremities, appearing within minutes of vigorous exercise (Figure 2a). The eruptions occurred daily, were consistently reproducible in association with exercise for more than eight months, and resolved within six to eight hours without bruising or residual hyperpigmentation. The hives were not triggered by passive warming, hot bathing, or sweating unrelated to exercise or spicy food. Treatment with second-generation H1-antihistamine (*per os* desloratadine 5 mg 1–2 tablets per day, half an hour before exercise) resulted in partial improvement. She reported that hives were attenuated when she ingested effervescent potassium chloride (1.56 g, approximately 20 mEq of potassium) during physical activity, corresponding to approximately 20 mEq of potassium and allowing rapid potassium replacement. Serum potassium concentrations measured before and during the cutaneous episode remained within the reference range (interval from 3,9 to 5,1 mmol/L); however, during exercise (8th minute), the concentration was at the lower end of that range. The apparent improvement after oral administration of effervescent potassium chloride (1.56 g) during 15–30 min of physical activity was based on the patient’s report and was not evaluated in a controlled comparison. This improvement should therefore not be interpreted as evidence of treatment efficacy. At a later follow-up (2025), the patient reported intermittent recurrences of hives, absent of exercise, after consuming carbohydrate-rich foods (Table 3). On the basis of the available findings, that latest eruption was considered most compatible with chronic inducible urticaria, although a specific subtype could not be established with certainty. Cholinergic urticaria was considered less likely because the lesions were not punctate and were not triggered by passive warming. Exercise-induced anaphylaxis and food-dependent exercise-induced anaphylaxis were considered less likely because wheals were not accompanied by respiratory, cardiovascular, gastrointestinal or other systemic manifestations. Adrenergic urticaria was also considered less likely because the characteristic pale halo surrounding the hives was absent. During the same year (2020), she experienced episodes of palpitations and several episodes of tachycardia-induced syncope, occurring in the absence of exercise. Dynamic 24 h ECG monitoring revealed ventricular and supraventricular ectopic beats and couplets. Neuromuscular symptoms worsened following administration of propafenone, a class Ic antiarrhythmic drug. To prevent sudden cardiac death due to life-threatening ventricular arrhythmias, an implantable cardioverter–defibrillator was placed in October 2020. Several family members reportedly experienced sudden unexplained death at a young age. Her medical history revealed multiple dysmorphic features during childhood, including small lower jaw (micrognathia), prominent forehead, otoclysis with low-set ears, residual camptodactyly after surgical separation of simple syndactyly between the 2nd and 3rd fingers and between the 3rd and 4th fingers, and bilateral finger and toe clinodactyly (Figure 2b,c). She also reported several orthodontic and surgical procedures for dental crowding, as well as delayed secondary dentition and caries due to enamel hypomineralization. At the age of 16, she developed recurrent episodes of muscle cramps and weakness affecting both the upper and lower extremities, triggered by thermal stimuli and vigorous exercise. Treatment with acetazolamide and daily potassium chloride supplementation was initiated. A muscle biopsy showed no inflammatory infiltrates or muscle fiber destruction. Mild learning difficulties and deficits in executive function and abstract reasoning were also noted.

ATS was diagnosed on clinical grounds because the patient exhibited findings across its three characteristic domains: recurrent episodes of muscle weakness beginning in adolescence; documented ventricular ectopy associated with palpitations and syncope; and multiple dysmorphic abnormalities, including micrognathia, low-set ears, clinodactyly, syndactyly, and camptodactyly. These findings were considered to fulfill the proposed phenotype-based criteria for a clinical diagnosis of ATS; however, the diagnosis was not molecularly confirmed. Exome sequencing identified no pathogenic or likely pathogenic variant in *KCNJ2* or *KCNJ5*. A heterozygous missense variant (rs1158151032; p.Tyr936Cys) was identified in *SYNE2*; this variant remains of uncertain significance. *SYNE2* is not an established ATS-associated gene, and this finding was not considered evidence of potassium-channel dysfunction or a molecular explanation for the patient’s phenotype. Accordingly, the case is described as clinically diagnosed, genetically unresolved ATS, with no molecular ATS subtype assigned. However, dysfunction of potassium channels in mast cells, smooth muscle cells, and cardiac and skeletal myocytes may lead to membrane depolarization and a reduction in resting membrane potential. This depolarization can trigger the opening of voltage-gated calcium channels, resulting in increased intracellular calcium levels, which may promote mast-cell degranulation (hives), muscle dysfunction (periodic paralysis), and cardiac arrhythmias. Genetic testing for myotonic dystrophy type 1 and type 2 was negative. No pathogenic variants were identified in the genes associated with MELAS (mitochondrial enceaphalomyopathy, lactic acidosis and stroke-like episodes) or MERRF (myoclonic epilepsy with ragged-red fibers). The patient also had childhood asthma with polysensitization to inhalant allergens and seasonal allergic rhinoconjunctivitis. An extensive immunological workup was unremarkable, including ANA, extractable nuclear antigen (ENA) screening, myositis-specific antibodies, anti-double-stranded DNA (anti-dsDNA), anti-voltage gated potassium channel (VGKC)-complex antibodies, acetylcholine receptor (AChR) antibodies, MuSK antibodies, anti-endomysial IgA, anti-tissue transglutaminase IgA, anticardiolipin (IgM and IgG), anti-cyclic citrullinated peptide antibodies, complement C3 and C4, rheumatoid factor (RF), and serum protein electrophoresis. Additional investigations were normal, including for electromyoneurography (EMNG), serology-to-titin or low-density lipoprotein receptor-related protein 4 (LRP4), fecal calprotectin, stool antigen testing for Helicobacter pylori, tumor markers, vitamin levels, and C1 inhibitor activity and concentration.

At the 2025 follow-up mentioned above, the patient retrospectively associated some recurrences of hives with carbohydrate intake in the absence of exercise. This temporal association was not documented using a food-and-symptom diary or controlled challenge, and serum glucose, insulin, and potassium were not measured during these episodes. Although a high carbohydrate intake can increase insulin secretion and promote intracellular potassium uptake, it is unknown whether a clinically meaningful potassium shift occurred in this patient at the time of the skin lesions. Exercise intensity, meal timing and composition, medications, sweating, ambient temperature, the rest phase after exercise, and spontaneous fluctuation were not controlled and may have influenced the reported episodes. Ion flux and intracellular calcium signaling contribute to mast-cell activation, and experimental studies indicate that selected potassium channels can modulate mast-cell secretion. Nevertheless, these effects are channel-, cell-, and context-dependent. Mechanisms described in electrically excitable skeletal or cardiac myocytes cannot be directly extrapolated to human cutaneous mast cells, and the direction of membrane-potential effects on calcium entry is not captured by a simple depolarization model. No functional evidence in this patient links ATS-related potassium-channel dysfunction, extracellular potassium changes, or the *SYNE2* variant to cutaneous mast-cell activation. The proposed connection should therefore be regarded solely as an untested hypothesis.

## 7. Patient Approach When a Chronic Urticaria-Associated Syndrome Is Suspected

When approaching a patient with CU, it is recommended to perform several basic tests including ESR, CRP, neutrophil count during acute attacks, fibrinogen levels, and peripheral eosinophil count. Based on the patient’s medical history, additional targeted investigations might also need to be performed [56,57,58,59,60,61,62].

In cryopyrinopathies, a detailed family history should be obtained as all syndromes in this group are inherited in an autosomal dominant pattern and check for cryoglobulins and causes thereof (electrophoresis of serum, infections, autoimmune connective tissue diseases, lymphoproliferation) [3,18]. Many autoinflammatory syndromes affect certain ethnic populations (e.g., FMF is most seen in individuals of Armenian, Arab, Turkish, or non-Ashkenazi Jewish descent; MKD predominantly manifests in Dutch and other northern European individuals) and have early onset (e.g., MKD usually presents during the first 6 months of life; NOMID during the first week of life). The duration, associated symptoms, and resolution of individual hives are the important clinical clues used to differentiate classic urticaria (hives) from other dermatological mimickers. In classic urticaria, individual pruritic hives are transient and resolve within 1 to 24 h without leaving residual post-inflammatory hyperpigmentation, while the urticaria associated with FCAS is delayed in its onset, usually several hours after exposure to cold, and persists for up to 48 h. Patients more commonly complain of burning or stinging than pruritus [3,18,41].

UV is distinguished from ordinary urticaria by the presence of hives that persist for longer than 24 h. This time threshold is the most reliable clinical indicator for distinguishing whether hives are an isolated symptom or part of a broader syndrome. Furthermore, skin lesions in UV resolve with residual purpura or post-inflammatory hyperpigmentation, due to erythrocyte extravasation [41,42]. Skin biopsy is an important component of the diagnostic work up of systemic urticarial syndromes and should be considered for hives that persist in the exact same spots longer than 24 h. Histopathological findings demonstrate dense, neutrophil-predominant inflammatory infiltrates in the dermis with leukocytoclasia. Neutrophilic urticarial dermatosis is an interstitial and perivascular neutrophilic infiltrate with leukocytoclasia, but without true vasculitic vessel-wall damage. This distinct pattern carries direct diagnostic significance confirming that the diagnosis is not an immune complex-mediated small-vessel vasculitis. The absence of immune-complex deposition and complement abnormalities further support the diagnosis of an autoinflammatory syndrome.

Physical causes of urticaria can be reproduced under controlled conditions to confirm a diagnosis (e.g., the ice cube test, when cold is the suspected trigger). The ice cube test (cold provocation test) is negative in CAPS/FCAS at the site of contact—although generalized cold exposure can trigger a rash—while in acquired cold urticaria it is positive, producing an immediate localized wheal/flare within minutes of direct contact and rewarming. Clinically, acquired cold urticaria involves classic itchy wheals restricted to localized areas exposed to cold (ice, cold wind, cold water, etc.). Rare cases of systemic/anaphylactic spread are seen when large areas of the body are exposed, e.g., when swimming. Cryopyrinopathies, however, involve maculopapular, urticarial-like systemic eruptions and are triggered by generalized environmental temperature drops, accompanied by systemic inflammation, fevers, and arthralgias [63,64].

In contrast to autoimmune diseases, which are mediated by the adaptive immune system, autoinflammatory disorders are diseases of the innate immune system. Autoantibodies (ANA, ENA, ANCA, dsDNA, RhF), immunoglobulins, and complement studies (C3, C4) are valuable diagnostic and prognostic markers if UV or Sjögren syndrome are suspected [40,42]. Monoclonal IgM gammopathy is a key diagnostic hallmark of Schnitzler syndrome, hypogammaglobulinaemia indicates PLAID, while elevated serum IgD and mevalonic acid in the urine are helpful biomarkers used to diagnose MKD, which help differentiate it from other periodic fever syndromes like FMF [10,35]. In CU patient management, the recognition of systemic manifestations associated with autoinflammatory syndromes is crucial for establishing a correct diagnosis and choosing an appropriate treatment. The periodicity of febrile attacks also has diagnostic value, e.g., febrile attacks in TRAPS tend to last longer (1–3 weeks) than those of FMF (24–48 h) or MKD (4–5 days) [7,19]. Additional clinical features may further suggest autoinflammatory syndromes, such as painful myalgias and serositis (peritonitis, pleuritis, and pericarditis) that precede the appearance of hives in TRAPS; sensorineural hearing loss in MWS; serositis (peritonitis, pleuritis, and synovitis) in FMF; painful cervical lymphadenopathy, hepatosplenomegaly and aphthous stomatitis in MKD, etc. [7,19]. As a group of conditions, these autoinflammatory disorders are often characterized by a delay in diagnosis of several years and an increased risk of severe, systemic AA amyloidosis, which primarily targets the kidneys and liver (e.g., FMF). When patients with CU present with systemic features such as periodic fevers, joint or muscle pain, organ involvement (serosal membranes, hepatosplenic, ocular or neurological) and elevated inflammatory markers, a structured diagnostic workup (including histopathological examination, serological evaluation and targeted genetic testing) is required to allow appropriate therapeutic intervention because urticaria associated with autoinflammatory syndromes fails to respond to H1 or H2 antihistamines because they are driven by IL-1 and other cytokine pathways rather than histamine.

Finally, the key steps to recognizing CU-associated syndromes are outlined below. They are based on a diagnostic algorithm for CU according to current EAACI guideline recommendations (Table 4) [56,57].

## 8. Conclusions

CU may occur as part of a broad spectrum of syndromic conditions. Therefore, it is essential to recognize associated clinical manifestations and investigate potential underlying syndromes, as establishing the correct diagnosis is crucial for appropriate patient management and treatment. Management of these conditions often requires a multidisciplinary approach involving dermatologists, rheumatologists, pediatricians, internists, and infectious disease specialists. To our knowledge, the case study included in this article is the first report of a co-occurrence of CU and ATS. Ultimately, distinguishing whether urticaria represents an isolated disorder or is a manifestation of a broader systemic syndrome is of critical importance, as this may significantly influence both diagnostic evaluation and therapeutic decision-making.

## Figures and Tables

**Table 1:** Key data useful for distinguishing the prominent types of conditions: (1.) true chronic spontaneous and chronic inducible urticaria; (2.) neutrophilic urticarial dermatoses associated with autoinflammation; (3.) eosinophilic, vasculitic, and hematologic urticaria-like eruptions; (4.) autoimmune diseases that may coexist with urticaria without constituting a defined “urticaria syndrome”.

1. TRUE CSU AND CHRONIC INDUCIBLE URTICARIA	2. NEUTROPHILIC URTICARIAL DERMATOSES ASSOCIATED WITH AUTOINFLAMMATION	3. EOSINOPHILIC, VASCULITIC, AND HEMATOLOGIC URTICARIA-LIKE ERUPTIONS	4. AUTOIMMUNE DISEASES THAT MAY COEXIST WITH URTICARIA WITHOUT CONSTITUTING A DEFINED “URTICARIA SYNDROME”
**True CSU:** Wheals and/or angioedema occur unpredictably without external physical triggers, lasting <24 h per individual lesion. **Chronic Inducible Urticaria:** Lesions are reproducibly provoked by definite physical stimuli (e.g., cold, pressure, stroking/dermographism) and confirmed via standard provocation tests.	**Clinical Presentation:** Painful or burning lesions that persist >24 h, frequently accompanied by systemic symptoms like fever, arthralgia, and leukocytosis. **Histopathology:** Dense dermal neutrophilic infiltrate without true leukocytoclastic vasculitis (no vessel wall necrosis or fibrinoid deposits). Often linked to autoinflammatory syndromes like Schnitzler or adult-onset Still’s disease.	**Urticarial Vasculitis (UV):** Individual lesions last >24 h, are often painful rather than itchy, and resolve with bruising or post-inflammatory hyperpigmentation. Biopsy shows leukocytoclastic vasculitis with fibrin deposits in vessel walls. **Eosinophilic/Hematologic Eruptions:** Prolonged wheals rich in eosinophils, or underlying hematologic malignancies presenting with urticaria-like paraneoplastic rashes. Diagnosis requires CBC and targeted biopsies.	**Clinical Context:** Conditions like Hashimoto’s thyroiditis, type 1 diabetes, or celiac disease may occur alongside true CSU due to shared immune dysregulation. **Differentiation:** They do not create a distinct “urticaria syndrome”; skin lesions still behave as standard mast cell-driven wheals (<24 h duration), but associated autoantibodies or organ-specific symptoms are identified via targeted blood screening rather than skin biopsy.

Abbreviations: CBC—complete blood count; CSU—chronic spontaneous urticaria.

**Table 2:** Monogenic autoinflammatory syndromes associated with chronic urticaria (based on references [2,8,20,21,22,23,24,25,26,27,28,29,30,31,32,33,34]).

Syndrome	Gene (Locus)	Inheritance	Urticaria Type	Fever (Y/N)	Histology	Laboratory Findings	Treatment (If Available)	Genetic Testing to Consider
Familial Cold Autoinflammatory Syndrome (FCAS1)—mild CAPS phenotype	*NLRP3* [20,21] (1q44)	Autosomal dominant [20]	Cold-induced, recurrent maculopapular/“urticaria-like” rash; onset 1–4 h after cold exposure; usually non-pruritic [20,21].	Yes—cold-triggered fever/chills accompany flares [20,21]	Perivascular/interstitial neutrophilic infiltrate with leukocytoclasia, without true vasculitis; ±eosinophils (neutrophilic urticarial dermatosis) [21]	Leukocytosis, neutrophilia, ↑ ESR/CRP/serum amyloid A (SAA) during flares; markers often normalize between attacks [21]	Anti-IL-1 therapies: anakinra, canakinumab, rilonacept; cold avoidance/protective clothing as adjunct [21]	Single-gene *NLRP3* sequence analysis (exon 3 hotspot)—Sanger sequencing; NGS sequencing if somatic mosaicism is suspected [21]
Muckle–Wells Syndrome (MWS)—moderate CAPS phenotype	*NLRP3* [21,22] (1q44)	Autosomal dominant [22]	Episodic urticarial skin rash, less strictly cold-dependent than FCAS; may occur spontaneously [21,22].	Yes—episodic fever with arthralgia [21,22]	Neutrophilic urticarial dermatosis pattern (perivascular neutrophils, leukocytoclasia, no vasculitis) [21]	Leukocytosis, neutrophilia, ↑ ESR/CRP/SAA, proteinuria and microalbuminuria (risk of amyloidosis) [2,21]	Anti-IL-1 therapies: anakinra, canakinumab, rilonacept [21]	*NLRP3* sequence analysis; NGS-based deep sequencing if Sanger-negative (somatic mosaicism) [21]
Neonatal-Onset Multisystem Inflammatory Disease/Chronic Neurologic Cutaneous and Articular Syndrome (NOMID/CINCA)—severe CAPS phenotype	*NLRP3* [21,23] (1q44)	Autosomal dominant; frequently de novo (germline or somatic) [21,23]	Neonatal-onset, persistent, migratory urticaria-like rash present from birth/early infancy [21].	Yes—recurrent fever with chronic systemic inflammation [23]	Neutrophilic dermatosis pattern (perivascular neutrophilic infiltrate, leukocytoclasia) as in the wider CAPS spectrum [21]	Leukocytosis, neutrophilia, ↑ ESR/CRP/SAA, proteinuria and microalbuminuria (risk of amyloidosis) [2,21]	Anti-IL-1 therapies: anakinra (favored for CNS penetrance), canakinumab; multidisciplinary supportive care [21]	*NLRP3* sequence analysis; standard Sanger sequencing is negative—NGS sequencing recommended (mosaicism reported in up to 70% of previously “mutation-negative” cases; reported vertical transmission cases) [21]
Familial Mediterranean Fever (FMF)	*MEFV* [24] (16p13.3)	Autosomal recessive [24]	Not a core cutaneous feature. Hallmark lesion is erysipelas-like erythema (ELE) of the lower leg/ankle; recurrent urticaria has been reported only rarely [25].	Yes—recurrent short febrile episodes [25]	Perivascular neutrophilic infiltration without vasculitis [25]	↑ ESR, CRP, SAA (especially during flares), leukocytosis, thrombocytosis, proteinuria; SAA monitored for amyloidosis risk [25]	Colchicine (lifelong, first-line and amyloidosis-preventive); anti-IL-1 agents (anakinra, canakinumab, rilonacept) [25]	*MEFV* single-gene sequence analysis first; periodic fever multigene panel or comprehensive genomic testing (exome sequencing) if inconclusive [25]
TNF Receptor-Associated Periodic Syndrome (TRAPS)	*TNFRSF1A* [26] (12p13.31)	Autosomal dominant [26]	Not the primary lesion. Characteristic finding is a migratory maculopapular rash that travels distally with myalgia (not itchy); urticarial rash (itchy) and erysipelas-like erythema on lower limbs are also reported [26].	Yes—prolonged episodes (longer than other periodic fevers), occurring every 4–6 weeks [26]	Non-specific perivascular infiltrate of lymphocytes and monocytes [26]	Leukocytosis with ↑ absolute neutrophil count, ↑ ESR/CRP, markedly ↑ SAA during flares, proteinuria [26]	Anti-IL-1 therapy (anakinra, canakinumab) first-line (~90% response); TNF inhibitor etanercept as an alternative in refractory cases; corticosteroids for acute attacks only [26]	*TNFRSF1A* single-gene sequence analysis (>99% detection); periodic fever multigene panel or exome/genome sequencing if phenotype is atypical [26]
Mevalonate Kinase Deficiency/Hyperimmunoglobulinemia D Syndrome (MKD/HIDS)	*MVK* [27] (12q24.11)	Autosomal recessive [27]	Not a core diagnostic feature. Polymorphic skin lesions accompany fever (erythematous macules and papules most common, nodules, urticarial, morbilliform); purpuric/vasculitic lesions reported in severe cases [28].	Yes—short, recurrent febrile episodes [27]	Variable, not specific [28]	↑ ESR/CRP/SAA, leukocytosis; persistently ↑ serum IgD, IgA (not diagnostic); ↑ urinary mevalonic acid, specifically during febrile episodes [29,30]	Anti-IL-1 therapy (canakinumab, anakinra) first-line; anti-TNF and IL-6 s line; short-term glucocorticoids in flares [30]	*MVK* gene all-exon sequencing (Sanger or NGS); if negative, CNV detection. NGS panel test in atypical autoinflammatory syndrome [30]
PLCG2-Associated Antibody Deficiency and Immune Dysregulation (PLAID)/Familial Cold Autoinflammatory Syndrome 3 (FCAS3)	*PLCG2* [31] (16q23)	Autosomal dominant [31]	Cold urticaria with erythema and pruritus, characteristically triggered by evaporative cooling (not direct cold contact); present from infancy.	No—not a recognized feature of PLAID	Perivascular and interstitial mast-cell infiltrate; degranulated after cold exposure [33]	Low serum immunoglobulins (IgM, IgA), reduced/dysfunctional B cells; positive antinuclear antibodies in up to two-thirds of patients [32]	Avoidance of evaporative-cooling triggers; antihistamines often provide only partial relief; antibiotic prophylaxis or IVIG determined by history of infections [8]	NGS of the *PLCG2* gene, sometimes supplemented by RNA sequencing or whole-genome sequencing when standard exon sequencing misses causative in-frame deletions [34]

Abbreviations: CAPS—Cryopyrin-Associated Periodic Syndrome; CINCA—Chronic Neurologic Cutaneous and Articular Syndrome; CNS—Central Nervous System; CNV—Copy Number Variant; CRP—C-Reactive Protein; ELE—Erysipelas-Like Erythema; ESR—Erythrocyte Sedimentation Rate; FCAS1—Familial Cold Autoinflammatory Syndrome 1; FCAS3—Familial Cold Autoinflammatory Syndrome 3; FMF—Familial Mediterranean Fever; HIDS—Hyperimmunoglobulinemia D Syndrome; IgA—Immunoglobulin A; IgD—Immunoglobulin D; IgM—Immunoglobulin M; IL-1—Interleukin-1; IL-6—Interleukin-6; IVIG—Intravenous Immunoglobulin; MKD—Mevalonate Kinase Deficiency; MWS—Muckle–Wells Syndrome; NGS—Next-Generation Sequencing; NOMID—Neonatal-Onset Multisystem Inflammatory Disease; PLAID—*PLCG2*-Associated Antibody Deficiency and Immune Dysregulation; SAA—Serum Amyloid A; TNF—Tumor Necrosis Factor; TRAPS—TNF Receptor-Associated Periodic Syndrome. ↑—increased.

**Table 3:** Chronological clinical course of disease in a patient with concurrent chronic urticaria and Andersen–Tawil syndrome.

Time/Age	Clinical Course, Investigations, and Interventions	Source of Information
**Childhood** **(age not specified)**	Characteristic craniofacial, dental, and limb abnormalities were present, including micrognathia, prominent forehead, low-set ears, clinodactyly, syndactyly, and camptodactyly. The patient underwent orthodontic and corrective surgical procedures for dental crowding and congenital digital abnormalities.	Retrospective patient history and available medical records.
**Age 16 years**	Recurrent muscle cramps and weakness affecting the upper and lower extremities began and were associated with vigorous exercise and thermal stimuli. Serum potassium concentrations were measured several times and remained within the reference range. Treatment with acetazolamide and daily potassium chloride was initiated. Muscle biopsy showed no inflammatory infiltrates or muscle-fiber destruction.	Retrospective patient history and medical records.
**Age 27 years**	Daily, reproducible pruritic wheals without angioedema appeared within minutes of vigorous exercise. Up to ten lesions, each measuring less than 5 cm, were distributed symmetrically over the upper and lower extremities and resolved within 6–8 h without bruising or residual hyperpigmentation. A second-generation H1-antihistamine produced partial improvement.	Retrospectively reported by the patient at presentation; lesion morphology documented photographically.
**Age 28 years**	The patient experienced palpitations, tachycardia-associated syncope, and ventricular arrhythmia. Twenty-four-hour electrocardiographic monitoring showed ventricular and supraventricular ectopic beats and couplets. Neuromuscular symptoms worsened after propafenone.	Contemporaneous cardiology records and patient history.
**Age 28 years**	The patient first presented to the authors’ clinic with recurrent exercise-associated wheals. An implantable cardioverter–defibrillator was placed because of the risk associated with ventricular arrhythmia.	Contemporaneous clinical and cardiology records.
**Age 29 years**	Serum potassium concentrations measured before and during the episode remained within the reference range, although the concentration during exercise was at the lower end of that range. The patient reported attenuation of the wheals after potassium chloride; this observation was not evaluated in a controlled comparison.	Laboratory record and retrospective patient report.
**Age 29 years** **(genetic assessment)**	The combination of episodic weakness, documented ventricular ectopy associated with palpitations and syncope, and characteristic dysmorphic abnormalities supported a clinical diagnosis of Andersen–Tawil syndrome. Exome sequencing identified no pathogenic or likely pathogenic variants in *KCNJ2* or *KCNJ5*. A heterozygous *SYNE2* missense variant was classified as a variant of uncertain significance and did not provide molecular confirmation. Investigations for alternative neuromuscular, mitochondrial, immunologic, and urticaria-associated conditions were unrevealing.	Contemporaneous clinical, genetic, and laboratory records supplemented by retrospective history.
**Age 33 years**	The patient associated some recurrences of wheals with carbohydrate-rich food, including episodes without exercise. No food-and-symptom diary, controlled challenge, or simultaneous glucose, insulin, and potassium measurements were available. The association was therefore considered hypothesis-generating.	Retrospectively reported by the patient.

Abbreviations: KCNJ2—potassium inwardly rectifying channel subfamily J member 2; KCNJ5—potassium inwardly rectifying channel subfamily J member 5; SYNE2—spectrin repeat containing nuclear envelope protein 2.

**Table 4:** Diagnostic algorithm for recognizing chronic urticaria-associated syndromes based on current recommendations for chronic urticaria.

Diagnostic Algorithm Useful for Recognition of Chronic Urticaria-Associated Syndromes		
INITIAL CLINICAL EVALUATION ↓	Detailed history:	Record disease onset, hives/wheals shape, size, frequency, and duration, presence of angioedema, family history, triggers, and associated systemic symptoms (fever, bone/joint pain).
	Physical examination:	Assess skin distribution and check for signs of dermographism or physical triggers.
BASIC DIAGNOSTIC WORKUP (CHRONIC SPONTANEOUS URTICARIA) ↓	Routine labs:	DBC, CRP, and/or ESR
	Avoid broad screening:	Guidelines advise against costly/extensive general screenings for hidden infections or causes unless indicated by patient history.
EVALUATION OF ASSOCIATED SYNDROMES AND DIFFERENTIALS	Only wheals (No angioedema)	Rule out urticarial vasculitis (via skin biopsy) and autoinflammatory disorders (e.g., Schnitzler’s syndrome or CAPS) if systemic symptoms are present (e.g., bone pain and high CRP/ESR)
	Only recurrent angioedema (No wheals)	Consider bradykinin-mediated conditions, including ACE inhibitor-induced angioedema or HAE, by testing C4 and C1-inhibitor levels/function.
	Chronic inducible urticaria (CIndU)	Use specific provocation testing (e.g., ice cube test for cold, exercise for cholinergic) and measure thresholds when physical triggers are suspected.

Abbreviations: CRP—C-reactive protein; DBS—Differential blood count; EAS—erythrocyte sedimentation rate; CAPS—Cryopyrin-Associated Periodic Syndromes; HAE—Hereditary Angioedema. ↓—further step.

## Data Availability

No new data were created or analyzed in this study. Data sharing is not applicable to this article.
